# Evaluation of Cow-Side Meters to Determine Somatic Cell Count in Individual Cow Quarter and Bulk-Tank Milk Samples

**DOI:** 10.3390/ani13132169

**Published:** 2023-07-01

**Authors:** Leslie A. Jacobsen, Ashley M. Niesen, Padraig Lucey, Heidi A. Rossow

**Affiliations:** Veterinary Medicine Teaching and Research Center, University of California, Davis, 18830 Road 112, Tulare, CA 93274, USA

**Keywords:** dry off, SCC estimate, intramammary infection

## Abstract

**Simple Summary:**

Choosing which dairy cow mammary quarters to treat with antibiotics at dry off is difficult because current somatic cell count meters have not been tested and compared using the same data and metrics. The objective of this study is to compare predictions of somatic cell counts by current meters to a reference standard. The RT-10 meter best predicted individual quarter SCC ≤ 200,000 cells/mL followed by the DSCC which also best predicted BTM SCC ≤ 100,000 cells/mL. Using these meters to select which quarters to treat will help decrease antimicrobial use.

**Abstract:**

Intramammary infections, which cause mastitis, can increase treatment and labor costs, decrease milk production, and affect milk quality. Meters that measure quarter somatic cell count (SCC) could be used to make more informed dry cow therapy decisions. The objective of this study was to compare the RT-10 iPhone adapter (RT-10; Dairy Quality Inc., Newmarket, ON, Canada), DeLaval Cell Counter (DSCC; DeLaval, Gurnee, IL, USA), Porta Check Quick Test (PortaCheck, White City, OR, USA), California Mastitis Test (ImmuCell, Portland, ME USA), pH meter (Hanna Instruments, Smithfield, RI, USA), electrical conductivity meter (OHAUS, Parsippany, NJ, USA), and the dual laser infrared temperature thermometer (Klein Tools, Lincolnshire, IL, USA) for measuring SCC in individual Holstein mammary quarters in comparison to a reference standard, the Fourier Transform Spectrometer 600 Combi System (Combi; Bentley Instruments, Chaska, MN, USA). Meters were evaluated using 658 individual cow quarter samples and 100 bulk-tank samples to measure SCC. Individual quarter milk samples from 160 cows from four commercial dairy herds were collected just before dry off and tested within 4 h of collection. To test bulk-tank SCC, 100 bulk-tank milk samples (25 mL) were collected from UC Davis Veterinary Medicine Teaching and Research Milk Quality Lab. Meter SCC values were regressed on observed Combi SCC. Goodness of fit was then evaluated by partitioning the mean square predicted error (MSPE). For individual quarter SCC, RT-10 had the highest coefficient of determination (R^2^ = 0.86), lowest MSPE, and highest proportion of MSPE due to random variation (96%). Both the RT-10 and DSCC had the highest sensitivity and specificity for identifying quarter SCC above and below 200,000 cells/mL. For bulk-tank SCC, DSCC had the highest coefficient of determination (R^2^ = 0.45), lowest MSPE, and highest proportion of MSPE due to random variation (80%). The RT-10 and DSCC could be used to measure individual quarter SCC to determine which cows to treat at dry off potentially reducing antibiotic use.

## 1. Introduction

Dairy producers have multiple treatment options at dry off to combat intramammary infections, such as the use of blanket and selective therapies. Blanket dry-cow therapy, the practice of treating every cow at dry-off with antibiotics, has been the most prominent dry-cow therapy used. In 2014, 80% of dairy herds (and 94.2% of large dairy operations) used this method [1]. However, only 35.6% of cows at dry-off may have a prevalence of intramammary infections [2]. Dairy producers may be unnecessarily treating cows with antibiotics, leading to increased treatment costs. Selective dry-cow therapy, treating only udders or quarters that are infected, is an alternative to blanket therapies [3]. This strategy has been shown to be effective in herds that already have a low risk of intramammary infection [4]. Blanket dry cow therapies have been found to be slightly more expensive than selective therapies, $54.7–$58.5 and $52.4–$58.2 per cow, respectively, and selective dry cow therapy showed a 29% reduction of antibiotic use in comparison to blanket therapy [5]. Selective dry-cow therapy has the potential to reduce the use of antibiotics used for dry-cows without adversely affecting milk production or health status.

Monitoring both individual cow somatic cell counts (SCC) and bulk tank milk (BTM) can provide an opportunity for dairy producers to improve milk quality, production, cow health, and lower treatment costs [6,7]. Commercially available cow-side meters can be quick and easy to use. Several studies have highlighted the need for tests that are sensitive for SCC detection, and that can be measured cow-side [8,9,10]. Several studies evaluated two to three cow-side tests that can measure SCC [11,12], measure udder temperature [13], esterase activity and pH [14], milk electrical conductivity [15,16], and SCC at previous test day [17], but few have compared all of these tests on milk samples from multiple herds.

The objective of this study was to compare and evaluate seven cow-side meters by using individual quarter milk samples obtained at dry-off and BTM samples in comparison to a reference standard, Combi. Milk samples from dry-off were chosen because antibiotic treatment decisions for selective or blanket therapies for the cow are made at this time.

## 2. Material and Methods

### 2.1. Experimental Design

An observational study was conducted using 40 Holstein cows per dairy from 4 commercial dairy herds (*n* = 658 quarters total) located in Tulare and Fresno County, California. Individual quarter milk samples were collected on the same day and just prior to drying off. Bulk-tank milk samples (*n* = 100) were provided by the UC Davis Veterinary Medicine Teaching and Research Center’s Milk Quality Laboratory. No knowledge of the cows, location of the dairy, or other management factors of these dairy herds were known, other than sample collection time (at AM or PM). Samples were collected between October 2020 and August 2021, and there were no exclusion criteria for individual or BTM samples.

Cows in Dairies 1 and 3 were housed in dry lots with headlocks. Cows in Dairies 2 and 4 were housed in free stall barns with headlocks. Dairies 1, 3, and 4 milked cows twice a day, whereas Dairy 2 milked cows 3 times a day. No procedures were undertaken that were different from the regular cow handling protocols of the dairies, and there were no humane endpoints for the cows on this study.

### 2.2. Individual Quarter and BTM Sampling

To collect each individual quarter sample, teat ends were wiped with ethanol-soaked gauze (3 mL), and foremilk was discarded. Fifty mL of milk was hand collected into separate tubes for each quarter. Milk samples were then placed on ice and transported to the lab. Then, 25 mL of milk were separated into two different vials, with 5 mL for analysis by DHIA using the Combi system and the rest for bench top analyses.

For BTM sampling, 50 mL of BTM milk were collected from the laboratory after bacteriological sampling had occurred. Twenty-five mL were aliquoted into separate vials, one for bench top analysis and the other for Tulare County Dairy Herd Improvement Association (DHIA). Only meters RT-10 adapter (RT-10; Dairy Quality Inc., Newmarket, ON, Canada), DeLaval Cell Counter (DSCC; DeLaval, Gurnee, IL, USA), PortaCheck Quick Test (PC; PortaCheck, White City, OR, USA), electrical conductivity meter (ECM; OHAUS, Parsippany, NJ, USA), and pH meter (HC; Hanna Instruments, Smithfield, RI, USA) were used for BTM analysis, as the samples were not obtained cow-side.

### 2.3. SCC Analyses

All individual quarter and BTM samples were mixed gently prior to measurement of SCC. The Combi, which was designated as the reference standard test, was owned and operated by DHIA, and was a combination of two modules, a Fourier Transform Spectrometer and a Flow Cytometer, for the determination of SCC and milk composition. Cells within the milk sample were stained with a fluorescent dye and exposed to a laser beam, and then light was refracted in accordance with the amount of somatic cells.

The DSCC was a portable machine that uses cassettes to predict SCC. The RT-10 for the iPhone 5/s used the same cassettes to predict SCC. The cassettes contained a fluorescent reagent (propidium iodide) to stain the cell’s nuclei. In the DSCC, the cassette’s counting window was exposed to a light emitting diode, a digital camera photographed a picture of the stained nuclei, and then the DSCC counted the nuclei. The DSCC had a measuring range of 10,000 to 4,000,000 somatic cells/mL. In the RT-10, the camera of the iPhone was used to count SCC. There can be a high rate of cartridge failure if the counting window was damaged or smudged when handled, which can affect the usefulness of the device.

The PC used test strips that estimated the number of somatic cells by measuring the esterase enzyme present within white blood cells. The test strip was mixed with milk and an activator solution [3-(*N*-tosyl-lalanyloxy)-indol] (~80 µL) in the sample well, which caused a color change to blue, and represented the amount of esterase in the sample. A color chart was used to score SCC results in categories of ≤100,000; 250,000, 500,000, 750,000, 1,500,000, ≥3,000,000 cells/mL.

The California Mastitis Test (CMT; ImmuCell, Portland, ME, USA) was a qualitative four-welled plastic paddle that can test a cow’s individual quarters for SCC. A reagent that broke down cell membranes and contained a pH indicator (bromocresol purple) caused the milk to gel in accordance with the concentration of SCC. Milk and reagent were added to the paddle and were rotated and tilted until the reaction was completed. Results were scored a Negative (<200,000 SCC), Trace (150,000–500,000 SCC), +1 (400,000–1,500,000 SCC), +2 (800,000–5,000,000 SCC), or +3 (>5,000,000 SCC) according to the standards set by the manufacturer. 

The ECM used electrodes to measure the resistance or density of milk, which was electrically positive. This model had a range of 0.00–19.99 S/m, and an accuracy of ±2.5% fs.

The HC used an electrode to measure the pH of milk. Changes in milk pH were due to compositional changes, such as extracellular fluid components and blood, which led to an increase in pH. This meter had a range of 0–14 pH, and an accuracy of ±0.05 pH.

Individual quarter temperatures were taken 1 h after sampling using a dual infrared temperature thermometer (IR5; Klein Tools, Lincolnshire, IL, USA). The device used dual laser beams as a focal point for the temperature sensor on the front part of the tool for individual quarter temperature determination. The emissivity level was set to 0.98, the emissivity value used to measure the temperature of human skin [18]. This meter was aimed at the caudal area of each quarter. Temperature readings were able to be taken from a safe distance (~1.5 m) from the cow due to the dual laser to lessen personal risk and cow stress.

A common practice for dairies to decide which cows to treat at dry-off is the previous month’s SCC (PSCC) [19] by using machines such as the Combi. However, if the days between the previous test date and the days since last test (DSLT) are far apart, PSCC may change as infections may be hard to detect in between the SCC test and the dry-off [20]. Dairy 1 PSCC and DSLT data were used to evaluate observed SCC because they had the lowest herd SCC and they treated all cows at dry-off.

### 2.4. Sample Size Determination

The minimum number of samples for regression was 8 [21]. This study used at least 40 cows per dairy as the sample size. Since the dairy and the cow were not significant contributor variables to the regressions, total sample size was 658 for individual quarter samples SCC and 100 for BTM SCC. No data points were excluded from the data analyses.

### 2.5. Statistical Analysis

The unit of interest in this study was mammary gland individual quarter and BTM sample SCC. We defined cell counts ≥ 200,000 SCC cells/mL as an intramammary infection based on [22]. Positive intramammary infection tests were also defined by manufacturer’s directions as SCC ≥ 200,000 cells/mL for DSCC and RT-10 meters, category ≥ Trace for CMT, SCC category ≥ 250,000 cells/mL for the PC meter. Positive intramammary infections were defined as ECM meter readings ≥ 5.0 mS/cm [16], pH ≥ 6.6 for the HC meter [23], Temperatures ≥ 35 °C for IR5 [24], and of SCC ≥ 200,000 cells/mL for PSCC [17].

To determine how well meters were able to predict individual quarter SCC measured by the Combi, SCC predicted by each meter was regressed on Combi SCC using general linear models [25]. The independent variable was Combi SCC, and each regression contained one of the dependent variables, RT-10, DSCC, PC, CMT, ECM, HC, IR5 SCC, dairy, and cow. Dairy and cow were not significant contributors to the prediction of SCC in any of the regressions, and the residual errors were normally distributed.

Dairy 1 was the only dairy that tested all cows using DHIA each month. So, to evaluate the practice of using previous test date SCC (PSCC), Combi SCC were regressed on DSLT and PSCC using Dairy 1 data only using general linear models [25]. Combi SCC had to be averaged by cow for each monthly test date since PSCC is a composite sample from the mammary gland. Since DSLT was not a significant contributor to Combi SCC, it was dropped from the regression, so the dependent variable was Combi SCC, with independent variable PSCC. 

To evaluate how well meters predicted BTM SCC, the SCC estimated by the meters RT-10, DSCC, ECM, and HC were each regressed on Combi SCC using general linear models [25]. The dependent variable was Combi SCC, and each regression contained one of the independent variables RT-10, DSCC, ECM, and HC SCC. 

For individual quarter and BTM meter predictions of SCC, tests for goodness of fit, and the coefficient of determination (R^2^), mean bias (MB%), error due to mean absolute error (MAE), and partitioning of the mean square predictive error (MSPE) due to central tendency (CT%), unequal variation (UEV%), error due to random variation (RV%), and error due to slope ≠ 1 (%) were evaluated [26].

Determination of diagnostic sensitivity (SE), specificity (SP), prevalence, accuracy, likelihood ratios positive (LR+), negative (LR−), and predictive values positive (PPV) and negative (NPV) for each meter for both individual quarter and bulk-tank milk samples was completed using a diagnostic test evaluation calculator [27]. Disease prevalence was calculated as the total number of positives using the Combi divided by the total number of milk samples. 

## 3. Results and Discussion

Detection of high SCC is needed to improve milk production, cow health, and, ultimately, reduce the use of antibiotics at dry-off. To reduce intramammary infections in herds, dairy producers can employ different meters that assess the amount of SCC in individual cow quarter and bulk-tank milk. This study compares the performance of several common SCC meters and PSCC to predict SCC at dry-off. 

The four dairies represented different herd management practices, with different herd sizes, milk yields, and milk components (Table 1). For example, due to the low price of pregnant heifers, Dairy 4 purchased pregnant heifers as replacements, and milked all cows as long as possible. Dairy 4 also did not subscribe to monthly milk testing, so no milk yield or milk composition data were available. They had the highest average DIM, DIM at dry-off, and lowest average parity. These management differences are also reflected in the descriptive statistics for Table 2. The average and standard deviation for SCC across these herds were high because Dairies 2, 3, and 4 did not monitor monthly SCC and Dairies 2 and 4 did not prophylactically treat their cows at dry off for high SCC. Dairy 1 treated all cows at dry-off, had the lowest average quarter SCC, lowest DIM at dry-off, lowest SCC, and relatively high milk production because reproductive management was a priority at this dairy. All dairies had 2–3 cows with excessively high SCC, in the range of 3,000,000 to 8,000,000 SCC. So, while most cows were below 200,000 SCC, there were 66 out of 659 quarters that were greater than 1,000,000 SCC. Days since last test (DSLT) was only available for Herd 1 and 3, since these were the only herds that measured SCC in their herd test. Using DSLT and SCC at last test to predict SCC at dry-off was only possible for Herd 1 because Herd 3 only measured SCC every 3 months. 

### 3.1. Performance of Meters on Individual Quarter Milk Samples

**Figure 1:**
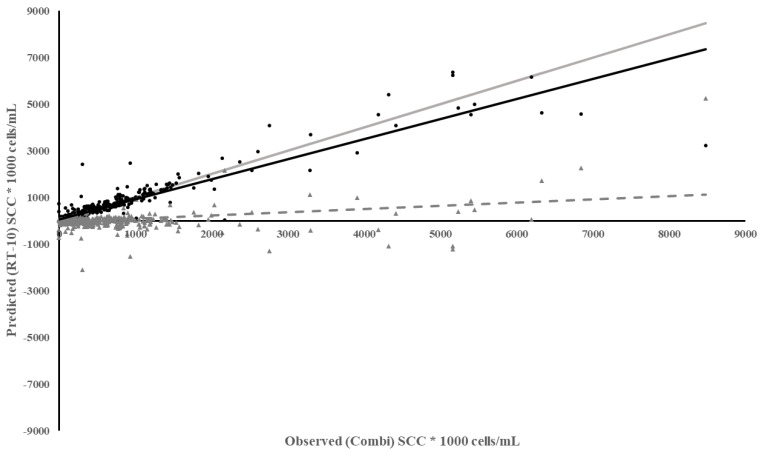
Plot of Predicted SCC using the RT-10 vs. Observed SCC from Combi based on individual milk samples. Meter Data (•), Residuals (▲), Regression (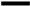), Reference Regression line (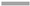) where slope = 1, intercept = 0, Regression of Residuals on Observed ( - - - - ) *p* < 0.01.

The RT-10 meter best predicted SCC in individual cow quarters compared to the other meters (Table 3). The regression of predicted RT-10 on observed Combi quarter SCC had a slope closest to 1, i.e., predicted equaling observed SCC, the highest coefficient of determination, and one of the lowest MSPE. The MSPE was split into three different categories of error: bias of prediction (difference contained within the model’s predicted values versus the observed), slope not equal to 1 (error associated with the slope not being equal to 1), and random variation (variation contained within the observed data). Most of the MSPE was due to random variation in the data. The residuals regressed on observed Combi quarter SCC had a low but consistent bias (Figure 1). The under prediction of SCC by the RT-10 had less of an impact when SCC were below 200,000 cells/mL. Therefore, the RT-10 was the best meter for measuring SCC below 200,000 cells/mL but, as SCC increased, the error associated with the measurement also increased. 

The RT-10 and DSCC had similar SE (Table 4), and compared to other SCC tests, they had a greater percent ability to identify a quarter with an infection (SCC > 200,000 cells/mL), SP, a greater percent ability to identify a quarter without infection, a greater percent ability to differentiate between SCC above and below 200,000 cells/mL, and, lastly, accuracy. Previous studies used the same level of 200,000 cells/mL to determine SE and SP for the RT-10 and DSCC. Compared to the previous study [11], the RT-10 and DSCC showed a greater ability to differentiate infection status in individual quarters and estimated an approximately 10% higher SE and 4% higher SP. 

**Figure 2:**
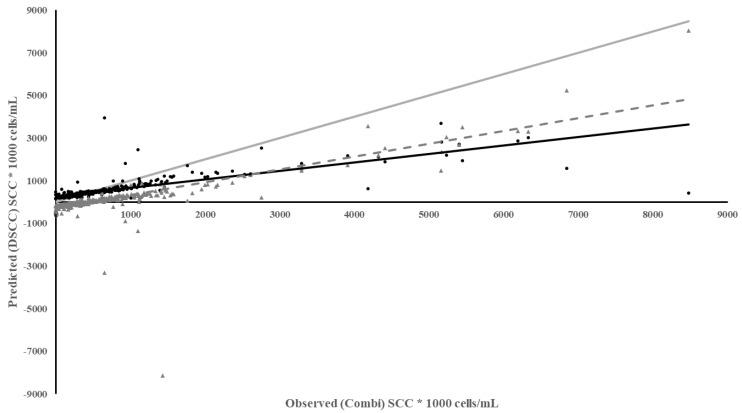
Plot of Predicted SCC using the DSCC vs. Observed SCC from the Combi based on individual milk samples. Meter Data (•), Residuals (▲), Regression (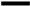), Reference Regression line (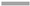) where slope = 1, intercept = 0, Regression of Residuals on Observed ( - - - - ) *p* < 0.01.

The DSCC meter and RT-10 meter used the same methods and cartridges to measure SCC. So, it would be expected that they would perform well. While the DSCC meter performed well in diagnostic measures, SE and SP, the regression of predicted DSCC on observed Combi quarter SCC had a larger intercept, much lower coefficient of determination, larger MSPE with a higher error due the slope not being equal to 1 (Table 3). The DSCC under predicted SCC to a greater extent especially above SCC of 100,000 cells/mL and the underestimation increased as SCC increased with a bias in the residuals (Figure 2). However, if milk lactose content was added to the regression of predicted DSCC on observed Combi SCC, the ability of the DSCC to predict SCC improved (R^2^ = 0.44). This implies that the difference between the performance of the two meters is due to a better camera in the iPhone as the DSCC may lose some resolution of SCC with more lactose in the milk. 

The PC meter was the third best at predicting SCC. This meter is a colorimetric meter only able to predict a range of SCC within specified SCC categories. So, the interpretation of results is not as precise as other meters since it relies on the ability of the user to match a color chart that designates a range of SCC values. The PC meter classifies SCC within these ordinal categories: ≤100,000, 250,000, 500,000, 750,000, 1,500,000, and ≥3,000,000 cells/mL. The predicted SCC by the PC meter vs. observed Combi SCC regression slope was further from unity than the RT-10 and DSCC and had a higher coefficient of determination and lower MSPE than the DSCC, while more of the proportion of error was due to random variation in the data (Table 3). The PC meter had a lower SE, SP, and accuracy compared to the RT-10 and DSCC (Table 4). Previous studies also used an SCC of 250,000 cells/mL to determine SE and SP, and estimated an 8% higher SE and 11% lower SP than in this study, possibly due to colorimetric SCC ranges being subjective. If a dairy were to use any of these meters to determine whether to treat a cow for mastitis, they should consider which meter will predict SCC well within their desired SCC cut-off to treat to decide which cows should be treated for their dry-cow treatment program. 

**Figure 3:**
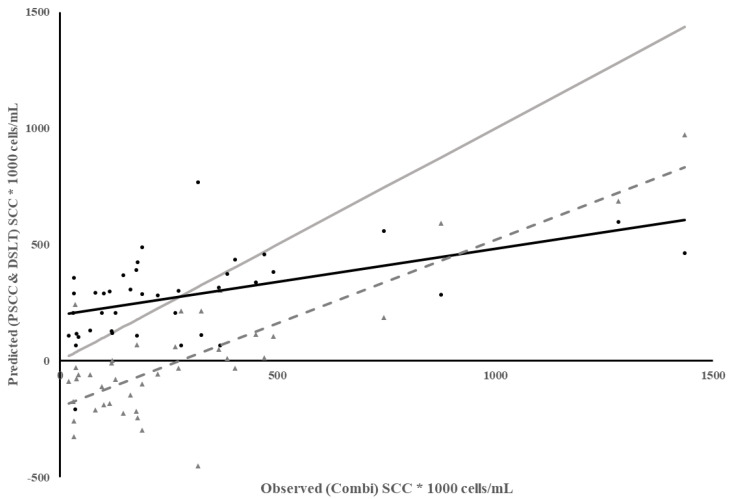
Plot of Predicted SCC using previous test day SCC (PSCC) vs. observed SCC from Combi for individual milk samples. Combi SCC from each quarter were averaged per cow according to each test day in Dairy 1. Meter Data (•), Residuals (▲), Regression (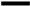), Reference Regression line (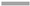) where slope = 1, intercept = 0, Regression of Residuals on Observed SCC using Combi ( - - - - ) *p* < 0.01.

Because many dairies use PSCC at the most recent DHIA test to identify cows to treat at dry-off, one more method of predicting SCC was evaluated. Since the Combi SCC was only measured on individual quarters, SCC from each quarter were averaged to estimate Combi SCC and compare to PSCC. The regression of PSCC on observed SCC from Combi had a low coefficient of determination and low MSPE but most of the error was due to a poor model fit, with high error due to slope not equaling 1 and bias in the predictions (Table 3). The predictability of Combi SCC using PSCC decreased as Combi SCC increased (Figure 3), indicated by a steady increase in the residuals as Combi SCC increased. This led to lower predictability and bias of prediction, which was not unexpected since infections that result in a high SCC for a short period of time may be harder to detect in-between monthly test days as these infections are associated with environmental pathogens [20]. 

The SE, SP, and accuracy of PSCC were also low compared to the other meters (Table 4). Compared to [17], SE was lower but SP was similar for the thresholds. This analysis suggests that PSCC was a poor predictor of average Combi SCC. However, using averaged individual quarter SCC from Combi may not be representative of SCC collected as a combined sample from the udder, particularly from udders with infected quarter. Typical monthly DHIA milk test samples are blended, and it is assumed that each quarter contributed an equal amount of milk to the blended sample. However, if one of the quarters has a high SCC, milk sample flow could change and would not contribute the same amount of milk to the blended sample. The blended sample would no longer represent SCC [30]. In this study, quarter infection status was different among the 40 enrolled cows from Dairy 1 with 30% of cows with an infection in all quarters (SCC > 200,000), 19% in three quarters, 23% in two quarters, and 26% in one quarter. If the thickness of the high SCC milk sample affected the SCC in the blended sample, the blended sample SCC will never represent the SCC of the cow and will not be representative of the level of mammary infection. This is particularly a problem when only one or two quarters are infected, which was almost 50% of the cows at Dairy 1.

### 3.2. Performance of Meters on BTM Samples

The meters RT-10, DSCC, PC, ECM, and HC were chosen for BTM analysis as these meters did not need to be used on fresh milk samples or in the presence of the cow. The DSCC meter and RT-10 meters best predicted BTM SCC with the highest coefficients of determination, lowest MSPE, and highest proportion of MSPE due to random variation (Table 5). Both the DSCC and RT-10 had the best SE, SP, and accuracy (Table 6). Compared to RT-10 and DSCC SE and SP in Table 4, SE was 6% higher and SP was 24% lower. The difference in performance of the two meters for BTM SCC might be due to the milk samples being older, since they were not measured until 12–24 h after collection from the bulk tank. The longer length of time may have affected the ability of the meters to estimate SCC. Settling of solids in the bulk tank may have also made it difficult to obtain uniform samples, and increased solids in the sample could interfere with meter function.

### 3.3. Meter Measurement Performance Comparison

The RT-10, PC, and DSCC meters were the best in their ability to predict SCC. The DSCC meter is large and heavy, costs $1960.22 more than the RT-10, and analyzes samples 10 s faster than the RT-10 (Table 7). The DSCC meter also performed worse at the prediction of individual quarter SCC compared to the RT-10. While the RT-10 performed the best at the prediction of individual quarter SCC, the adapter is only fitted for the iPhone 5s, and has not been updated for newer versions of the iPhone, and nor has it been made available for the android phone. The RT-10 uses a Dairy Health Management application for record storage, which makes it easy to use compared to the DSCC. The management application allows users to collect and store SCC and cow health data, input cow numbers, and create and export excel spreadsheets with health and SCC data. For SCC analysis, the DSCC and the RT-10 can both be used cow-side or in a laboratory setting. The cartridges for both the DSCC and RT-10 cost about $2.10 per quarter, with producers able to test one cow for about $8.40 compared to $1.00 per quarter for DHIA samples using the combi. Since dry-cow treatments generally cost $5.00 per quarter, if a dairy is willing to selectively treat individual quarters within cows, using the meter could save $11.60 per cow not treated. 

The PC test strip is available in different versions, one being a 45 min version and the other being a 5 min version, with both being able to be used on-farm. The 5 min version was used in this study because 45 min was too long to wait for results. There could be missed treatments in the dry-off period as the threshold for the detection of SCC is <150,000 cells/mL for this meter, and it did not perform as well as SCC increased. The test kit includes 20 pouches with two test strips in each pouch, 10 pipettes, activator solution, and instructions on how to perform the test, which cost $1.01 per sample, with all four quarters testing for about $4.04. While this test can be used cow-side, it is more feasible to perform this test on a lab benchtop. Results from [14] were similar, although they also tested the 45 min version and found it had higher SE and SP. 

The meters CMT, ECM, HC, and IR5 performed poorly on the analysis of individual quarter and BTM samples compare to the RT-10, DSCC, and PC. While the CMT can be used as an indicator for high SCC, this meter had very low SP for high SCC in this study. There is also an element of human error with the CMT, as not every person will read the results in the same way [31]. The ECM and HC could measure samples quickly; however, ECM had low SP and HC had low SE high SCC. These results were similar to [16,23] for ECM and milk pH, respectively. The IR5 was easy to use cow side but had the lowest SE for measuring high SCC in our study. It was the suggestion in [24] that infrared thermography was sensitive enough to pick up changes in skin temperature but was probably not useful to confirm an intramammary infection. However, they used the CMT as the gold standard for comparison, which is not a quantative test for SCC, and which they also found to be difficult to interpret.

## 4. Conclusions

Meters that can accurately measure individual mammary quarter SCC have the potential to reduce antibiotic treatment of non-infected quarters and to decrease treatment costs associated with dry-cow therapies. The RT-10 meter best predicted individual quarter SCC ≤ 200,000 cells/mL followed by the DSCC which also best predicted BTM SCC ≤ 100,000 cells/mL. It may be beneficial for dairies to use these cow-side meters instead of PSCC. As PSCC increases, predictability and the bias of prediction to underpredict SCC increases.

## Figures and Tables

**Table 1:** Differences in herd management practices and milk production parameters of dairies enrolled.

Dairy Sampled ^1^	Total Cows Milking	Average DIM	DIM at Dry-Off	Average Parity	305 ME kg	SCC × 1000	Milk Fat %	Milk Protein %	Times Milked
1	1471	197	335	1.9	14,300	281	5.72	3.45	2
2	4000	190	340	2.4	14,700	551	5.32	3.57	3
3	2500	143	340	2.3	11,800	546	4.13	3.82	2
4 ^2^	2732	318	430	2.1					2

^1^ Dairies 1, 2, and 4 used dairy herd management software DairyComp 305 (Tulare, CA, USA), and Dairy 3 used dairy herd management software DHI-Plus (Provo, UT, USA). Data for Dairies 1, 2, and 3 were obtained from monthly DHIA herd testing records. ^2^ Dairy 4 does not employ monthly milk testing.

**Table 2:** Dry-cow management practices of cows enrolled at each dairy.

Dairy Sampled ^1^	Pen Type ^2^	Season Sampled	Average DSLT (SD) ^3^	Average Parity (SD)	Average DIM at Dry-Off (SD)	Average DCC (SD) ^4^	Average SCC × 1000 (SD)	Times Sampled	Quarters Sampled
1	DL	Fall	15 (8)	1.9 (1.3)	287 (31)	224 (1)	282 (499)	7:00 AM	160
2	FS	Winter		2.0 (1.1)	233 (57)	233 (7)	551 (967)	8:00 AM	176
3	DL	Summer	85 (9)	1.8 (1.0)	337 (75)	236 (2)	546 (1129)	7:30 AM	162
4	FS	Summer		1.7 (1.1)	336 (93)	203 (1)	337 (646)	7:00 AM	160

^1^ Dairies 1, 2, and 4 used dairy herd management software DairyComp 305 (Tulare, CA, USA), and Dairy 3 used dairy herd management software DHI-Plus (Provo, UT, USA). ^2^ Pen types where cow quarter milk samples were taken are dry lot (DL) or free stall (FS) using composted manure. ^3^ Average days since last test (DSLT) and Standard deviation (SD). ^4^ Average days carried calf (DCC).

**Table 3:** Regression of meter SCC values on observed SCC (Combi) from individual cow quarters (*n* = 658).

Descriptive Statistics ^1^	DSCC ^2^	RT-10	PC ^3^	CMT	ECM	HC	IR5	PSCC ^4^ *n* = 40
Observed mean, SCC × 1000	432	432	432	432	432	432	432	282
Predicted mean ^5^, SCC × 1000	432	432	250	400	573	435	432	281
Observed SD, SCC × 1000	859	859	859	859	859	859	859	316
Predicted SD, SCC × 1000	543	797	641	87	3203	38	0.240	139
Linear Regression ^6^								
Intercept	178	−28.8	−79.3	570	−749	0	431	186
Slope	0.60	1.1	1.5	−56	210	64	0.010	0.49
Mean Square Error (MSE)	62,806	102,106	66,829	190,505	312,121	198,002	197,907	82,463
Coefficient of Determination (R^2^)	0.40	0.86	0.56	0.010	0.080	0.0040	0.0	0.19
Mean Bias, %	−0.030	0.030	0.15	−0.11	−33	−0.48	0.030	0.35
Mean Absolute Error (MAE)	251	111	259	436	559	445	445	182
Mean Square Predicted Error (MSPE)	442,154	101,685	326,367	729,200	10,986,013	733,223	736,542	78,341
Partition of MSPE, %								
Error due to bias of prediction	3	1	0	3	0	0	1	0
Error due to slope ≠ 1	23	4	14	82	50	92	99	61
Error due to random variation	77	96	86	18	50	8	0	39

^1^ Fourier Transform Spectrometer 600 Combi System (Combi; Bentley Instruments, Chaska, MN, USA) values were regressed on SCC predictions by the other meters using PROC GLM in SAS [25]. ^2^ DeLaval Cell Counter (DSCC; DeLaval Gurnee, IL, USA), RT-10 iPhone adapter (RT-10; Dairy Quality Inc., Newmarket, ON, Canada), PortaCheck Quick Test (PC; PortaCheck, White City, NJ, USA), California Mastitis Test (CMT; Dairy Research Products, Inc., Eden Prairie, MN, USA), electrical conductivity meter (ECM; OHAUS, Parsippany, NJ, USA), pH meter (HC; Hanna Instruments, Smithfield, RI, USA), dual laser infrared temperature thermometer (IR5; Klein Tools, Lincolnshire, IL, USA). ^3^ Meters PC and CMT are discontinuous tests, as they only predict ranges of SCC. The largest range number that corresponded to the color change of the test were chosen as the values of SCC. ^4^ Previous test day SCC (PSCC) regression was completed using data from Dairy 1 as this dairy recorded PSCC, and individual quarter samples for the Combi were averaged by test month for the regression analysis. ^5^ Meters PC and CMT are discontinuous tests, and the predicted means were chosen using average SCC range values of the meters. ^6^ Goodness of fit was evaluated according to [26].

**Table 4:** Tests of sensitivity, specificity, prevalence, and accuracy with confidence intervals of predictions for meter estimations of SCC in individual cow quarters.

Diagnostic Test ^1^	DSCC ^2^	RT-10	PC	CMT	ECM	HC	IR5	PSCC ^3^
SE, %	92.5	91.5	74.0	97.6	86.4	26.8	0	67.2
95% CI	(88.9–95.3)	(84.7–94.4)	(69.1–78.7)	(95.2–99.0)	(82.0–90.1)	(21.8–32.2)	(0–1.24)	(54.3–78.4)
SP, %	90.1	90.4	89.7	16.3	33.9	69.2	100	74.5
95% CI	(86.5–93.0)	(86.9–93.2)	(85.7–92.9)	(12.6–20.5)	(29.0–39.0)	(64.1–73.9)	(0–0)	(64.7–82.8)
Prevalence ^4^, %	50.3	50.2	59.0	44.8	81.3	61.9	44.8	54.9
Accuracy, %	91.2	90.9	81.2	52.7	57.5	50.2	55.2	71.6
LR+, %	9.3	9.5	7.2	1.2	1.3	0.87	0	2.6
LR−, %	0.080	0.090	0.29	0.15	0.40	1.1	1	0.44
PPV, %	88.4	88.5	89.5	48.7	51.5	41.4	0	63.2
NPV, %	93.7	92.9	74.4	89.4	75.5	53.8	55.2	77.7
Previously Published								
SE, %	82.0	82.0	81.0	57.4	43.5	83.0	95.6	86.0
SP, %	86.0	86.0	78.0	72.3	92.9	29.0	93.6	50.0
References	[11]	[11]	[14]	[28]	[15]	[25]	[13]	[29]

^1^ Sensitivity (Se), specificity (Sp), lower and upper bounds of the 95% CI, accuracy, positive (LR+) and negative (LR−) likelihood ratios, and positive (PPV) and negative (NPV) predictive values were calculated by using the diagnostic test evaluation calculator by [27]. ^2^ DeLaval Cell Counter (DSCC; DeLaval Gurnee, IL, USA), RT-10 iPhone adapter (RT-10; Dairy Quality Inc., Newmarket, ON, Canada), PortaCheck Quick Test (PC; PortaCheck, White City, OR, USA), California Mastitis Test (CMT; Dairy Research Products, Inc., Eden Prairie, MN, USA), electrical conductivity meter (ECM; OHAUS, Parsippany, NJ, USA), pH meter (HC; Hanna Instruments, Smithfield, RI, USA), dual laser infrared temperature thermometer (IR5; Klein Tools, Lincolnshire, IL, USA), Days since last test PSCC was previous SCC. This diagnostic was completed using only data from Dairy 1 as this dairy recorded PSCC. DSCC, RT-10, CMT, ECM, HC, and IR5, and PSCC used SCC 200,000 cells/mL and above as positive infection. PC used 250,000 cells/mL and above as a positive infection. ^4^ Prevalence was calculated by the total number of positives divided by the total number of milk samples.

**Table 5:** Statistical summary of results from regression of predicted on observed bulk-tank SCC values (*n* = 0).

Descriptive Statistics ^1^	DSCC ^2^	RT-10	PC ^3^	ECM	HC
Observed mean, SCC × 1000	182	182	182	182	182
Predicted mean, SCC × 1000	182	182	100	182	182
Observed SD, SCC × 1000	70	70	70	70	70
Predicted SD, SCC × 1000	40	36	19	12	10
Linear Regression ^4^					
Intercept	92.9	109	137	211	−118
Slope	0.40	0.31	0.26	−7.1	41.5
Mean Square Error (MSE)	1834	1983	2970	3408	3406
Coefficient of Determination (R^2^)	0.33	0.26	0.08	0.03	0.02
Mean Bias, %	−4.0	−2.5	1.8	−6.8	1.2
Mean Absolute Error (MAE)	43	45	54	58	58
Mean Square Predicted Error (MSPE)	3200	3546	4426	4663	4700
Partition of MSPE, %					
Error due to bias of prediction, %	2	6	3	3	1
Error due to slope ≠ 1, %	27	32	57	72	75
Error due to random variation, %	73	68	43	28	25

^1^ DeLaval Cell Counter (DSCC; DeLaval Gurnee, IL, USA), RT-10 iPhone adapter (RT-10; Dairy Quality Inc., Newmarket ON, Canada), PortaCheck Quick Test (PC; PortaCheck, White City, OR, USA), California Mastitis Test (CMT; Dairy Research Products, Inc., Eden Prairie, MN, USA), electrical conductivity meter (ECM; OHAUS, Parsippany, NJ, USA), pH meter (HC; Hanna Instruments, Smithfield, RI, USA), dual laser infrared temperature thermometer (IR5; Klein Tools, Lincolnshire, IL, USA). ^2^ Combi values were regressed on SCC predictions by the other meters using [25]. ^3^ Meter PC is a discontinuous test, as it only predicts ranges of SCC. The largest range number that corresponded to the color change of the test was chosen as the value of SCC. ^4^ Goodness of fit were evaluated according to [26].

**Table 6:** Tests of sensitivity, specificity, prevalence, and accuracy with confidence intervals of predictions for meter estimations of SCC in bulk tank milk.

Diagnostic Test ^1^	DSCC ^2^	RT-10	PC	ECM	HC
SE, %	85.7	88.1	59.5	21.4	0
95% CI	(71.5–94.6)	(74.4–96.0)	(43.3–74.4)	(10.3–36.8)	(0.00–100)
SP, %	70.7	62.1	58.6	62.1	100
95% CI	(57.3–81.9)	(48.4–74.5)	(44.9–71.4)	(48.4–74.5)	(93.8–100)
Prevalence ^3^, %	59	64	66	64	42
Accuracy, %	77	73	59	45	58
LR+, %	2.9	2.3	1.4	0.56	0
LR−, %	0.20	0.19	0.69	1.3	1.0
PPV, %	68	63	51.	29.	0
NPV, %	87	88	67	52	58

^1^ Sensitivity (SE), specificity (SP), lower and upper bounds of the 95% CI, accuracy, positive (LR+) and negative (LR−) likelihood ratios, and positive (PPV) and negative (NPV) predictive values were calculated by using the diagnostic test evaluation calculator by [27]. ^2^ DeLaval Cell Counter (DSCC; DeLaval Gurnee, IL, USA), RT-10 iPhone adapter (RT-10; Dairy Quality Inc., Newmarket, ON, Canada), PortaCheck Quick Test (PC; PortaCheck, White City, OR, USA), electrical conductivity meter (ECM; OHAUS, Parsippany, NJ, USA), pH meter (HC; Hanna Instruments, Smithfield, RI, USA). ^3^ Prevalence was calculated by the total number of positives divided by the total number of milk samples.

**Table 7:** Comparison of ease of use and costs of meters.

	Combi ^1^	DSCC	RT-10	PC	CMT	ECM	HC	IR5
Meter cost ^2^, $	350,000	3497	1533	40	15	61	150	60
Cost per sample ^3^, $	1.00	2.10	2.10	1.01	0.04	0	0	0
Measurement time ^4^	7 s	50 s	60 s	5 m	10 s	10 s	30 s	2 s
Volume of milk sampled	6 mL	60 µL	60 µL	80 µL	3 mL	10 mL	10 mL	0
Sample environment	Lab	Both	Both	Lab	Cow-side	Lab	Lab	Cow-side

^1^ A FTS 600 (Combi; Bently Instruments, Chaska, MN, USA), RT-10 iPhone adapter (RT-10; Dairy Quality Inc., Newmarket, ON, Canada), DeLaval Cell Counter (DSCC; DeLaval Gurnee, IL, USA), PortaCheck Quick Test (PC; PortaCheck, White City, OR, USA), California Mastitis Test (CMT; Dairy Research Products, Inc., Eden Prairie, MN, USA), electrical conductivity meter (ECM; OHAUS, Parsippany, NJ, USA), pH meter (HC; Hanna Instruments, Smithfield, RI, USA), dual laser infrared temperature thermometer (IR5; Klein Tools, Lincolnshire, IL, USA). ^2^ Meter cost and volume of milk sampled was determined by the set cost and directions for use from the manufacture. ^3^ Cost per sample was calculated by dividing Meter Cost ($) by the number of samples included in the kits. ^4^ Measurement time was determined when the meter’s reading became consistent.

## Data Availability

The data was not deposited in an official repository but is available upon request.
